# Visits to Private Asylums

**Published:** 1903-09-12

**Authors:** 


### VISITS TO PRIVATE ASYLUMS.

By Our Rambling Reporter.

### BROOK VILLA, GREEN LANE, LIVERPOOL.

This private asylum is situated near the centre of a very large population, being not more than three miles from the Lime Street Station; and it can be easily reached in about twenty minutes by electric tram-car from St. George's Hall, as the line passes within a hundred yards or so of the villa. The gates stand open, and a nice avenue winds up to the main entrance. The grounds attached to the asylum and belonging to it are 20 acres in extent, and are tastefully laid out and planted. Although so near a large city and so conveniently placed, the building is sufficiently secluded to prevent its being overlooked too much.

The house is licensed to accommodate 52 patients, and both sexes are admitted. At present the asylum is almost full. Some of the patients are on parole and are allowed to wander about unattended. There are a great many one-bedded rooms in the house; in fact, fully two-thirds of the patients have their own bedrooms, and this is an arrangement which is nearly always liked by patients themselves, and is approved of by the relatives. The asylum does not contain one large dormitory, but there are several which contain two or three beds, and, when occasion requires the presence of an attendant or nurse during the night, these rooms can be called into use. As there are no large dormitories, neither are there any large sitting-rooms, and the small rooms have, in most instances, a very home-like appearance. The dining-room is, however, a large and handsome apartment, and here the ladies and gentlemen have their meals together, the table being presided over by one of the medical officers. There is a good billiard-room. The whole building is very comfortable throughout. The rooms are warmed by open fireplaces, assisted when low temperature requires, by hot-water radiators.

Of late years the recoveries seem to have reached nearly 50 per cent. of the admissions—a very creditable percentage when it is remembered that nearly all classes of the insane have been admitted. The rates of payment range from £2 to £3 a week, and some have been received at even less than £2. The proprietors are Drs. Duffus and Cooke, and at least one of them is always in residence.

This may be named as another of our private asylums which should have power granted to it to considerably, or even greatly, to increase its numbers. Liverpool has nearly 600,000 inhabitants, and there is no other licensed house in the neighbourhood. Brook Villa should, therefore, be allowed to double, or perhaps treble, the number named in its license at present.

Hitherto no one, perhaps, has thought of Argentina in connection with Antarctic enterprise. Its comparatively close propinquity to the South Pole has induced the Argentine Government to endeavour to relieve the Nordenskjold party. The equipment provided is very complete, and, so far as clothing, bedding, and boots are concerned, has been entrusted to the Jaeger Company, who have already fitted out so many similar expeditions. Commander Irizar, Naval Attaché to the Argentine Legation in London, has started for Buenos Ayres to take over the command of the expedition.

